# Utility of Tonico-Astringent Injections into a Suppurative Abscess

**Published:** 1841-05

**Authors:** Norvin Green

**Affiliations:** Bedford, Ky.


### Art. II.—*Utility of Tonico-astringent Injections into a Suppurating Abscess. A Case. By* Norvin Green, M. D. *of Bedford, Ky.*

Mrs. W——, a stout, healthy female, aged 22, with a temperament partaking somewhat more of the lymphatic than the nervous, was delivered of her first born February 11th. On the 14th the left mamma presented symptoms of inflammatory action. Pain, hardness and swelling, were experienced, confined exclusively to the one side. Vinegar evaporations, light-bread and flax-seed poultices, and other well known and fashionable remedies were speedily resorted to; but without even a palliating effect. Drawing the breast and general reduction, however, were unfortunately neglected. The action proceeded rapidly to suppuration, and pointed on the 19th, externally and near to the nipple. From this time for more than three months, various applications in the form of poultices, salves, strong oozes, etc., had been successively employed, which, only serving to promote the rapidly wasting suppuration, it was deemed commendable to have medical aid.

On the 24th of May I was summoned to Mrs. W. Though quite alarmed at the stubbornness of her disorder, yet my patient was entirely ignorant of the amount of disorganization already effected by this draining malady: for almost the entire gland had been broken down and borne off by the ravages of unsparing suppuration. In three directions sinuses were formed: left and downwardly, right and downwardly, and almost directly upward. In the first mentioned opening, the probe passed with great facility to the distance of four inches; and in the last two, to nearly the same extent. The constitution, too, had participated largely in the suffering. The patient had greatly diminished in flesh and strength; stomach and bowels much disordered; a considerable degree of nocturnal restlessness, with strong threatening of, if not actually already, incipient hectic.

Such constitutional remedies, as were thought indicated by the general symptoms, were administered, and as nourishing a regimen adopted as the chylopoietic derangement would allow. A seton of linen tape was introduced through the left lateral sinus, existing at the base of the gland; and another, in like manner, through that of the right. Finding it next to impossible to prevail on my patient to submit to the pain of further operation, I consented to leave the diseased action of the superior portion unaddressed by local remedial agent, until some benefit should be realized from those already applied.

Due attention was given to the progress of the case; the setons were every day required to be moved. Fearing the tapes were insufficiently exciting, after a short trial they were rubbed, before moving, in pulvis plumbi acetas; and, finally, various mineral astringents were successively employed, (some by injection) to aid in exciting granulations. This course was perseveringly pursued for near six weeks, (the period allotted for a cure by Dr. Physic,) without sufficient benefit to encourage the hope of a reproduction of the parts, or the restoration of the organ to its natural function.

I now thought of laying open the abscess in the several directions of its ravages, and healing from the bottom as an ulcer; but the reluctance of my patient suspended the operation, until, in a correspondence with Prof. Miller, I enquired what more efficient applications would be worthy of a trial. Among other means, he recommended the use, by injection into the sinuses, of the *tinctura g. aloe et myrrhæ.* Entertaining the highest opinion of the practical skill, as well as scientific attainments of Dr. Miller, I was readily persuaded to give a fair trial to any remedy he might suggest. The tincture was accordingly prepared, after the formula of the Ed. Pharmacopœia, the saffron excepted. Its use was commenced on the 1st July, diluted, at first, to one part of the tincture to two parts of water, and the abscess directed to be filled twice in twenty-four hours; the injection to be retained as much as could be with a tent of lint, till the period of the succeeding dressing. The injection was ordered to be gradually increased in strength; but the thoughtful care of the patient did not allow its use at more than half strength, finding that, in an undiluted form, it produced painful and prejudicial irritation.

On the 10th July, I found the cure promisingly commenced, the sinuses beginning to fill up, and that one in farthest advance in which no seton had been introduced. The tapes were now removed, and all treatment suspended, save the injection, which the lady had learned to regulate to the best advantage. I saw the patient no more, but to furnish additional supplies of the remedy, which were as often sent by a messenger without further direction.

The recuperative powers now proceeded even more rapidly than had done the disorganising; it required successively a less amount of the tincture, than at the preceding dressing, to fill the cavity, till, by the 10th of August, the cure was complete. I have examined the breast, and find that the reparation is entire, and to all appearances, has every promise of the same usefulness as the other.

*Remarks.*—Great access to the cause of humanity was claimed, in the treatment of mammary abscess with the seton, as instituted by the "father of American surgery," in lieu of the "bold incision;" but, while I would not depreciate a popular remedy (supported, as this has ever been, by the highest authority,) on account of a single failure, yet it will be readily admitted, that should general experience corroberate the observations of this case, we have a class of remedies which need be attended with comparatively little inconvenience or suffering, and are far more efficient than even the boasted reformation. In this case, the superior efficacy of the vegetable tonic, *locally applied*, over mechanical irritation, or mineral astringents, for the purpose of changing an indolent and wasting into a granulating and repairing surface, is clearly exhibited; and it remains to be seen, whether the same will not obtain in ulcers and other lesions of this low and obstinately indolent character.

*Bedford, Ky.* 1840.

